# Editorial: Robust Artificial Intelligence for Neurorobotics

**DOI:** 10.3389/fnbot.2021.809903

**Published:** 2021-12-16

**Authors:** Joe Hays, Subramanian Ramamoorthy, Christian Tetzlaff

**Affiliations:** ^1^Robotics and Machine Learning Section, Dynamics and Control Systems Branch, Spacecraft Engineering Division, United States Naval Research Laboratory, Naval Center for Space Technology, Washington, DC, United States; ^2^Institute of Perception, Action and Behaviour, School of Informatics, University of Edinburgh, Edinburgh, United Kingdom; ^3^Bernstein Center for Computational Neuroscience, Third Institute of Physics, Georg-August-Universität Göttingen, Göttingen, Germany

**Keywords:** robotics, adaptation, neuromorphic, artificial intelligence, autonomy

## Introduction

Neural computing is a powerful paradigm that has revolutionized machine learning. Building from early roots in the study of adaptive behavior and attempts to understand information processing in parallel and distributed neural architectures, modern neural networks have convincingly demonstrated successes in numerous areas—transforming the practice of computer vision, natural language processing, and even computational biology.

Applications in robotics bring stringent constraints on size, weight and power constraints (SWaP), which challenge the developers of these technologies in new ways. Indeed, these requirements take us back to the roots of the field of neural computing, forcing us to ask how it could be that the human brain achieves with as little as 12 watts of power what seems to require entire server farms with state of the art computational and numerical methods. Likewise, even lowly insects demonstrate a degree of adaptivity and resilience that still defy easy explanation or computational replication.

**Figure 1:**
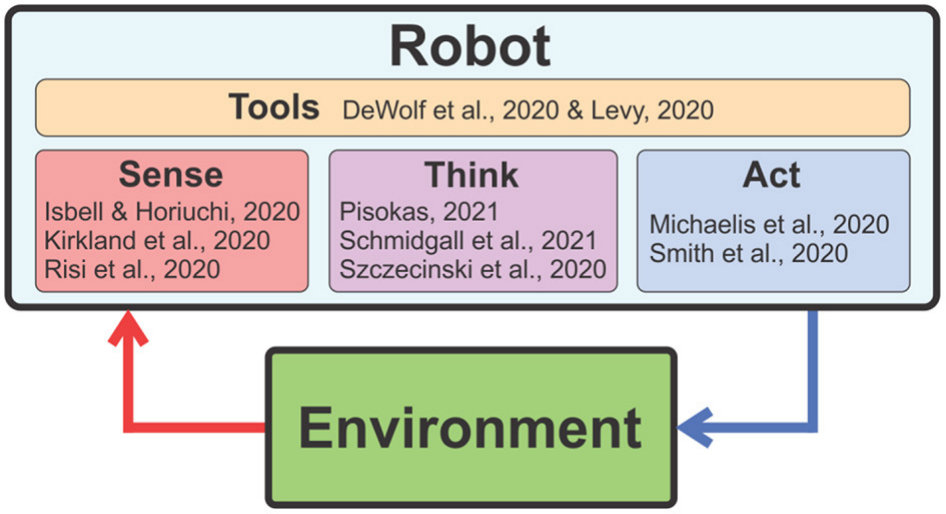
The articles of this Research Topic are organized into the following four themes: Sense, Think, Act, and Tools.

In this Research Topic, we have compiled the latest research addressing several aspects of these broadly defined challenge questions. As illustrated in Figure 1, the articles are organized into four prevailing themes: Sense, Think, Act, and Tools.

## Overview

### Sense

Three contributed articles focused primarily on the perception tasks of a robotic system. Specifically, Kirkland et al. investigated how to add understanding to the perception action cycle with spiking neural network (SNN) based segmentation. They demonstrated that an event based neuromorphic camera coupled with a Spiking fully Convolutional Neural Network (SpikeCNN) successfully provided semantic segmentation and understanding of their test scenes, and whose output was fed into a spiking control system providing actions.

Risi et al. presented a neuromorphic architecture for stereo vision, based on SNNs, that leverages the advantages of brain-inspired neuromorphic computing by interfacing two event-based vision sensors to an event-based mixed-signal analog/digital neuromorphic processor. Their results show a path toward the realization of low-latency, end-to-end event-based, neuromorphic architectures for stereo vision.

Finally, Isbell and Horiuchi presented the concept of Echo View Cells which used bat-inspired sonar to mimic how bats might sense objects in the environment and recognize the views associated with different places. They successfully demonstrated spatial invariance by training feed-forward neural networks, both traditional artificial neural networks (ANNs) and SNNs, to recognize 66 distinct places in a laboratory environment over a limited range of translations and rotations.

### Think

Three articles focused on general approaches to algorithm development based on Spiking Neural Networks, representing the thinking aspects of systems. Pisokas presented the perspective that the combination of reverse engineering with simulations allows the study of both the structure and function of biological neural circuits. This approach augmented understanding of both the computation performed by the neuronal circuit and the role of its components. Thus, a robotics practitioner can gain added inspiration and guidance in the development of network-based robotic algorithms.

Szczecinski et al. extend their previously-developed method for tuning ANNs, the “Functional Subnetwork Approach,” to SNNs based on generalized linear integrate-and-fire neurons. This extension enabled specific functions to be realized in SNNs through a direct analytic method of assembling and tuning the networks without the use of global optimization, or other forms of machine learning. Robotic algorithm developers are now able to directly implement specific functions in a network without training.

Finally, Schmidgall et al. introduced a framework, SpikePropamine, for simultaneously learning the underlying fixed-weights, and the rules governing the dynamics of synaptic plasticity and neuro-modulated synaptic plasticity in SNNs through gradient descent. This offers practitioners an approach to leverage the strengths of online plasticity in their robotic algorithm development through methods such as reinforcement learning.

### Act

Two articles addressed the action, or motor output, aspects of an embodied robotic system. Michaelis et al. presented a network architecture including the anisotropic network and a pooling layer which allows fast spike read-out from a neuromorphic processor (Intel's research chip Loihi) and performs an inherent regularization. With this, they showed that the anisotropic network reliably encoded sequential patterns of neural activity, each representing a robotic action, and that the patterns allowed the generation of multidimensional trajectories on control-relevant timescales.

Additionally, Smith et al. present latest results from their framework for self-organizing robotic sensorimotor control, DIAMOND, which employs a deep recurrent neural network, based on the principles of predictive coding. Their results provide evidence that deeper networks enable more complex exploratory behaviors.

### Tools

From a development tool perspective, Levy presented a minimalist application programming interface (API) for sensors and PID controllers, which makes it relatively easy for engineers to prototype neuromorphic approaches to micro-air-vehicle sensing and navigation.

Additionally, DeWolf et al. demonstrated how the Nengo neural modeling and simulation libraries enable users to quickly develop robotic perception and action neural networks for simulation on neuromorphic hardware. Hereby, users can rely on tools they are already familiar with, such as Keras and Python.

## Outlook for the Future

A long standing open question at the intersection of many fields—Artificial Intelligence, Neural Computing, Neuromorphic Systems and other forms of Biomimesis—pertains to the specification of how artificial systems should emulate the natural phenomena around learning and adaptation, and what the construction of such artificial systems might tell us about nature itself. Papers in this volume explore exactly this interface. With rapid advances both in our understanding of models (natural and artificial) and in our ability to fabricate new devices, the gaps between these diverse methodologies are rapidly closing, potentially enabling entirely new ways of answering these long-standing questions.

## Author Contributions

All authors reviewed multiple contributed articles in this research task and collaboratively wrote this Editorial.

## Conflict of Interest

The authors declare that the research was conducted in the absence of any commercial or financial relationships that could be construed as a potential conflict of interest. The Intel Corporation did not influence the current work nor had any role in it.

## Publisher's Note

All claims expressed in this article are solely those of the authors and do not necessarily represent those of their affiliated organizations, or those of the publisher, the editors and the reviewers. Any product that may be evaluated in this article, or claim that may be made by its manufacturer, is not guaranteed or endorsed by the publisher.

